# Successful expansion of functional and stable regulatory T cells for immunotherapy in liver transplantation

**DOI:** 10.18632/oncotarget.6927

**Published:** 2016-01-17

**Authors:** Niloufar Safinia, Trishan Vaikunthanathan, Henrieta Fraser, Sarah Thirkell, Katie Lowe, Laura Blackmore, Gavin Whitehouse, Marc Martinez-Llordella, Wayel Jassem, Alberto Sanchez-Fueyo, Robert I. Lechler, Giovanna Lombardi

**Affiliations:** ^1^ MRC Centre for Transplantation, Division of Transplantation Immunology and Mucosal Biology, King's College London, Guy's Hospital, London, UK; ^2^ Institute of Liver Studies, King's College Hospital, London, UK

**Keywords:** regulatory T cells, tolerance, immunotherapy, immunosuppression, liver transplantation, Immunology and Microbiology Section, Immune response, Immunity

## Abstract

Strategies to prevent organ transplant rejection whilst minimizing long-term immunosuppression are currently under intense investigation with regulatory T cells (Tregs) nearing clinical application. The clinical trial, ThRIL, recently commenced at King's College London, proposes to use Treg cell therapy to induce tolerance in liver transplant recipients, the success of which has the potential to revolutionize the management of these patients and enable a future of drug-free transplants. This is the first report of the manufacture of clinical grade Tregs from prospective liver transplant recipients via a CliniMACS-based GMP isolation technique and expanded using anti-CD3/CD28 beads, IL-2 and rapamycin. We report the enrichment of a pure, stable population of Tregs (>95% CD4^+^CD25^+^FOXP3^+^), reaching adequate numbers for their clinical application. Our protocol proved successful in, influencing the expansion of superior functional Tregs, as compared to freshly isolated cells, whilst also preventing their conversion to Th17 cells under pro-inflammatory conditions. We conclude with the manufacture of the final Treg product in the clinical research facility (CRF), a prerequisite for the clinical application of these cells. The data presented in this manuscript together with the much-anticipated clinical results from ThRIL, will undoubtedly inform the improved management of the liver transplant recipient.

## INTRODUCTION

Liver transplantation remains the treatment of choice for patients with end stage liver disease. Therapeutic advances in immunosuppression have led to a dramatic improvement in allotransplantation, averting acute rejection and supporting short-term graft survival. However, the obligatory protracted use of powerful non-specific immunosuppressants is complicated by increased morbidity and mortality as a result of chronic rejection and associated toxicity. The constant proportion of transplanted organs lost each year necessitating re-transplantation, in a climate of donor organ shortage, places further strain on an already saturated transplant waiting list.

The current approach to immunosuppression in transplantation is far from ideal with an enormous interest in the minimization/complete withdrawal of these drugs in liver transplant recipients. There have also been reports of a variable proportion of liver transplant recipients developing a state of ‘operational’ tolerance thus forgoing the requirements of therapeutic immunosuppression [1]. This phenomenon, however, only occurs late after transplantation and in a minority of patients [2]. It is, therefore, necessary to find novel strategies to allow for the development of tolerance early after transplantation and in turn negate the use of lifelong immunosuppression.

In this setting regulatory T cells (Tregs) are attractive candidates for therapeutic strategies aimed at tolerance induction, bearing in mind their integral role in promoting immune homeostasis. Characterised by the high and stable expression of surface interleukin-2 receptor α chain (IL-2Rα, CD25^hi^) and the transcription factor, FOXP3, these cells only constitute approximately 1-3% of circulating CD4^+^ T cells in the periphery [3-5]. As such, recent advances permitting the expansion of these cells *ex vivo* presents an attractive opportunity in modulating immune responses through their adoptive transfer. We have shown that infusion of recipient murine Tregs, expanded *in vitro*, can prolong skin allograft survival and induce indefinite acceptance of heart allografts [6]. More recently, the adoptive transfer of polyclonal human Tregs in humanised mice protected from human skin pathology and induced increased survival of transplanted islets [7-9].

The posited implications of Treg cell therapy in the context of liver transplantation have been aptly construed in murine models whereby liver allografts from tolerant mice were infiltrated with Tregs, and the depletion of these cells resulted in a loss of tolerance [10]. This circumstance is further mirrored in human subjects with increased frequencies of Tregs reported in operationally tolerant liver transplant recipients [11] paralleled by low circulating levels during acute rejection [12]. As such, there is little doubt that Tregs are promising candidates for tolerance induction in liver transplantation.

To date no clinical trials addressing the safety and efficacy of Treg cell therapy in the induction of tolerance in solid organ transplantation have been described. Recent trials have primarily focused on the safety profile of Treg therapy in the setting of bone marrow transplantation [13-15] and type I diabetes [16], with encouraging reports of efficacy. As a result, the prospects of applying Treg adoptive cell therapy in organ transplantation are now widely recognised.

In this regard, we have just opened the first combined Phase I/IIa clinical trial of Treg immunotherapy in the setting of liver transplantation, ThRIL (NCT02166177). Here, the safety, tolerability and efficacy of polyclonally expanded autologous Tregs from patients with end-stage liver disease, in combination with thymoglobulin and an mTOR-inhibitor-based immunosuppression regimen, will be assessed.

This is the first report, employing a Good Manufacturing Practice (GMP)-compliant protocol for the *ex vivo* expansion of Tregs from prospective liver transplant recipients. We further present the successful manufacture of the final drug product in the Biomedical Research Centre Clinical Research Facility (BRC CRF) at Guy's Hospital (King's College London) as a prelude for ThRIL.

## RESULTS

### Isolation and expansion of a pure Treg population from ARC patients

**Figure 1:**
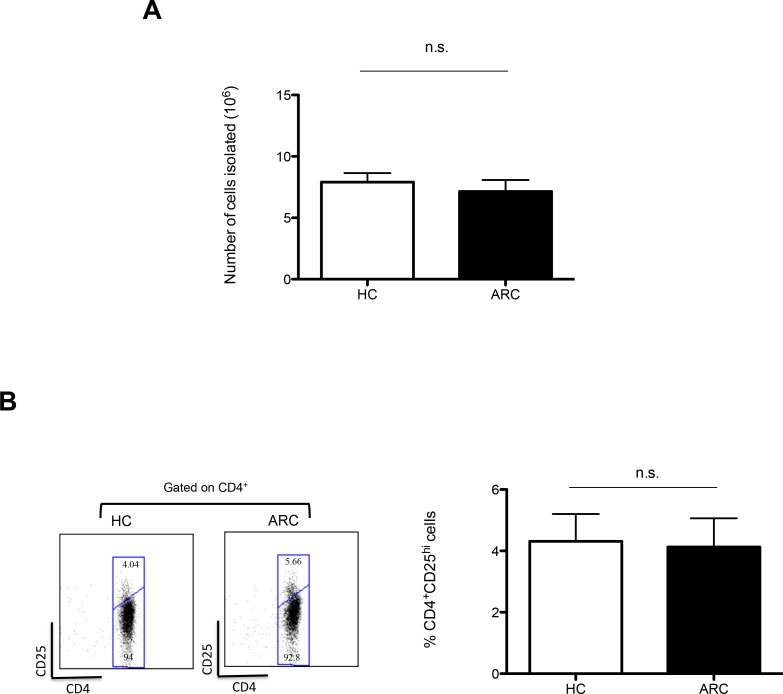
GMP Treg Isolation **A.** Numbers of cells isolated from 150ml of blood by CD8^+^ cell depletion and CD25^+^ cell enrichment compared between 9 ARC patients and 9 HCs. ***P < 0.001. **B.** Representative dot plot and graph denoting the circulating percentage of CD4^+^CD25^Hi^ Tregs of 5 ARC patients and 5 HCs. Abbreviation: n.s- not significant. Data are represented as mean +/− SEM.

In preparation for ThRIL, clinical grade autologous Tregs were isolated from 9 patients with alcohol related cirrhosis (ARC), awaiting transplantation, and 9 age and sex matched healthy controls (HCs). Using a protocol previously reported [17], similar numbers of cells were isolated from ARC patients and HCs (7.14×10^6^ ± 0.938 *vs*. 7.91×10^6^ ± 0.728, respectively; *P* = 0.528) (Figure 1A). This result confirms the similar frequency of Tregs observed *ex vivo* in the blood of ARC patients and HCs (4.13 ± 0.932 compared with 4.31 ± 0.889, respectively; *P* = 0.888) (Figure 1B).

The feasibility of autologous T cell therapy depends on success in the expansion of sufficient cell numbers *in vitro*. In this regard, Tregs were expanded using anti-CD3/CD28 monoclonal antibody-coated beads, high dose IL-2 and rapamycin, as previously reported [17, 18].

**Figure 2:**
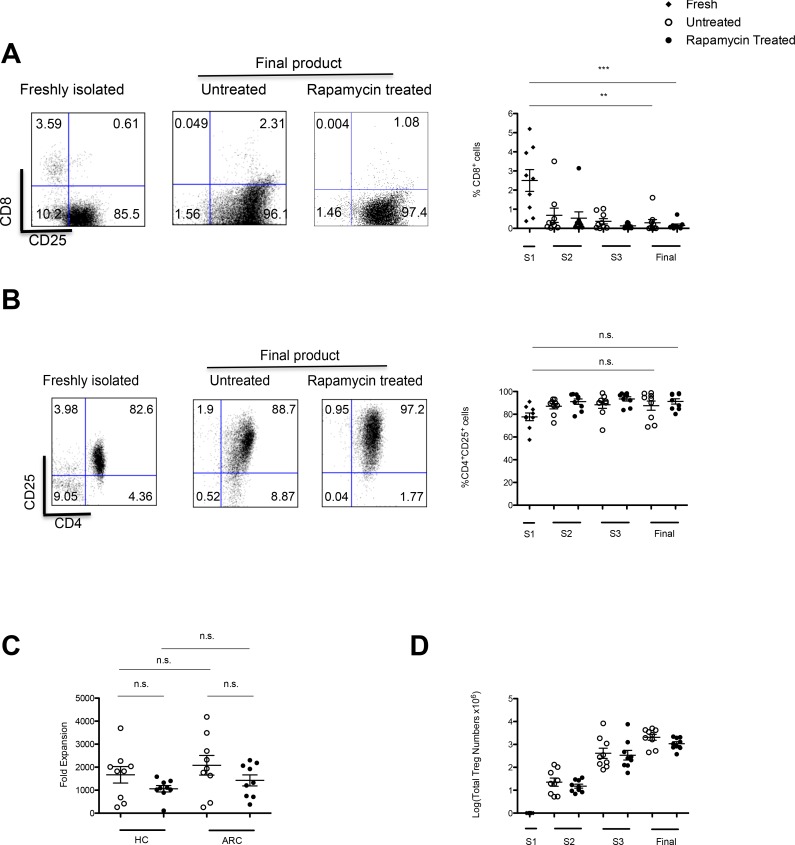
GMP Compatible Treg isolation and expansion Two step strategy for Treg isolation: **A.** CD8^+^ cell depletion. **B.** CD25^+^ cell enrichment. In each case dots plots are representative of 9 ARC patients. Graphs denote the purity of the culture throughout the expansion period in both rapamycin and untreated cultures **C.** Tregs from ARC patients and HCs were expanded over 36 days ± rapamycin. Fold expansion was calculated from Treg numbers at each stimulation. **D.** Predicted Treg numbers over the 36 day expansion period calculated based on fold expansion and the assumption that all cells were expanded at each stimulation. *n = 9 ARC, n = 9HCs.* Abbreviation: S, stimulation; S1, day 0; S2, day 12; S3, day 24; final, day 36; n.s, not significant. **P < 0.05, ***P < 0.001. Data are represented as mean +/− SEM.

Assessment of the percentages of cells expressing CD8^+^, CD4^+^ and CD25^+^ molecules, demonstrated on average a purity of 77.7% ± 10.3 CD4^+^CD25^+^ and 2.50 % ± 1.71 CD8^+^ cells at the start of the culture (S1). Expansion *in vitro* under the conditions described led to an enrichment of CD4^+^CD25^+^ cells; 91.3% ± 2.33, *P* = 0.004 with only 0.153% ± 0.073; *P* = 0.008 CD8^+^ cells at the end of culture (Figure 2A, 2B). The comparable purities of cells cultured in the absence of rapamycin were 87.5% ± 4.12; *P* = 0.088 and 0.292% ± 0.172; *P* = 0.0019, respectively.

In line with previous reports [19] the mean fluorescent intensity (MFI) of CD25 expression was highest following exposure of Tregs to rapamycin (data not shown).

### Tregs from ARC patients can be expanded to clinically suitable numbers

We next determined whether patient-derived Tregs could be expanded *in vitro* to numbers required for the maximum dose of Treg injection planned for ThRIL (4.5×10^6^/Kg).

Tregs from both patients and HCs expanded rapidly with comparable fold-expansion during the 36 days of culture (in the presence of rapamycin: ARC 1430 ± 239 *vs* HC 1060 ± 139; *P* = 0.207, in the absence of rapamycin: ARC 2080 ± 428 *vs* HC 1670 ± 359; *P* = 0.469) (Figure 2C). In addition, despite a trend for a reduced fold expansion there was no statistical difference in fold-expansion of Treg lines in the presence of rapamycin as compared to untreated cultures for both cohorts (ARC; 0.199, HC; *P* = 0.135). Additionally the average expansion of the 9 different Treg lines in the presence of rapamycin was 1.07 × 10^9^ cells ± 0.085 (Figure 2D), demonstrating the feasibility of reaching numbers needed for the high dose of Tregs planned to be administered in ThRIL.

### Rapamycin expanded Tregs maintained high levels of FOXP3, CD127lo and CTLA4 with a sustained expression of CD62L and CXCR3

**Figure 3:**
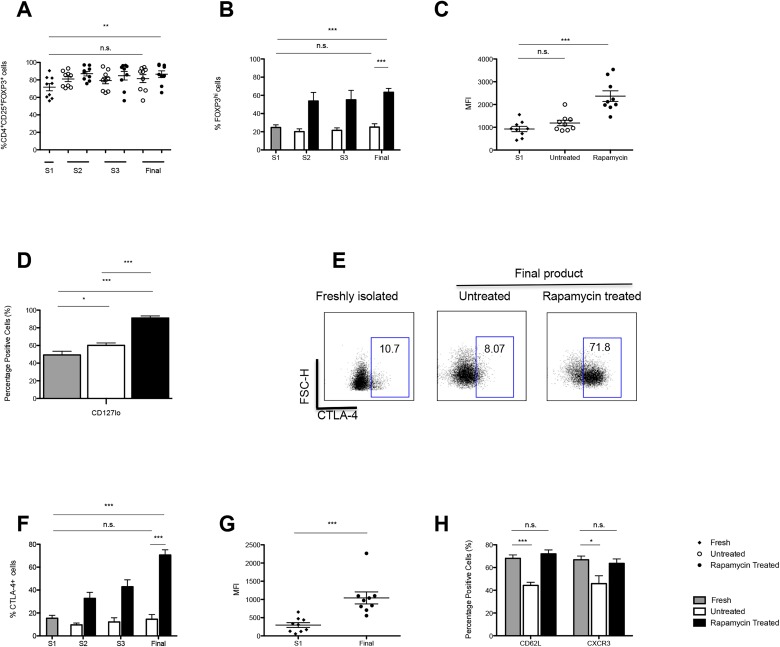
Expression of regulatory markers and homing receptor expression by Tregs throughout culture **A.** Graph shows the mean percentage of CD4^+^CD25^+^ cells expressing FOXP3^+^ in both untreated and rapamycin treated cultures over 36 days of culture (*n* = 9 ARC). **B.** Graph shows the frequency of FOXP3^Hi^ Tregs, from 9 ARC patients, throughout culture ± rapamycin. **C.** MFI of FOXP3 expression by Tregs. **D.** Graph shows the frequency CD127lo Tregs, from 9 ARC patients, throughout culture ± rapamycin. **E.** Dot plot details the frequency of CTLA-4 expression on CD4^+^CD25^+^ Tregs from one representative sample. **F.** The graph depicts the frequency of CD4^+^CD25^+^CTLA-4^+^ Tregs throughout culture ± rapamycin. G. MFI of CTLA-4 expression at S1 and day 36 of culture ± rapamycin. H. Expression of CD62L and CXCR3 on CD4^+^CD25^+^ on day 0 and at day 36 Tregs cultured ± rapamycin. Abbreviations: Mean fluorescent intensity (MFI) S- stimulation, n.s.-not significant. **P < 0.01, ***P < 0.001. Data are represented as mean +/− SEM. *n* = 9 ARC.

Having established the enrichment of CD4^+^CD25^+^ Tregs under these culture conditions, the expression of the transcription factor, FOXP3, of importance in Treg development and function was analysed [20]. Initially, upon isolation the total percentage of CD4^+^CD25^+^FOXP3^+^ cells stood at 77.7% ± 3.35, and contrary to published data reporting a loss of FOXP3 expression with prolonged periods of culture [21], the data reported an increase in the purity of culture in the presence of rapamycin to 90.66% ± 2.19, *P* =0.0053. (Figure 3A).

In addition, using a more stringent gating strategy whereby we were able to distinguish two distinct populations based on FOXP3 expression, termed FOXP3^Hi^ and FOXP3^Int^ (Supplementary Figure 1) it was also shown that the percentage of CD4^+^CD25^+^FOXP3^Hi^ cells was increased in culture in the presence of rapamycin (S1: 24.7% ± 2.95 *vs*. Final harvest: 63.4% ± 4.23; *P* < 0.0001) as compared to the untreated cultures (Figure 3B). In agreement, the MFI of FOXP3 was higher following exposure to rapamycin as compared to untreated cultures and baseline (Figure 3C).

Additionally, several subsets of Tregs have been described to date with reports that CD4^+^CD25^hi^ FOXP3^+^ Tregs typically lack the expression of the interleukin (IL)-7 receptor alpha chain, CD127 [22]. The differential expression of CD127 has been used to denote an optimally pure population of Tregs that is inversely correlated with FOXP3 levels and the suppressive function of human CD4^+^ Tregs. In support of the increase in purity of the cultures in the presence of rapamycin, our data demonstrate an increase in the frequency of CD127lo in the rapamycin treated cultures as compared the untreated cultures (*P* = <0.0001) (Figure 3D).

Constitutive high level of expression of CTLA-4 represents another well-documented trait of Tregs that has also been shown to contribute to their suppressive function [23-25]. Analysis of the cultures at final harvest revealed that rapamycin led to an increase in the percentage of Tregs expressing CTLA-4 (S1: 15.3% ± 2.51 *vs* Final harvest: 70.6% ± 4.522; *P* < 0.0001) as compared to untreated cells (Final harvest: 14.6 ± 4.15; *P* = 0.859) (Figure 3F) and this was mirrored by an increase in the MFI of this marker in rapamycin treated cultures as compared to baseline (Figure 3G).

An additional consideration regarding Treg therapy is the site of action of Tregs and, consequently, the desired homing properties of the injected cells. In the transplant setting, Treg lymph node homing and their ability to traffic to the graft are both required for their protection against graft rejection [26]. In this regard, it was shown that culture of Tregs in the presence of rapamycin maintained their expression of the lymphoid homing receptor, CD62L (S1: 68.1% ± 3.00 *vs*. Final harvest: 72.0% ± 3.51; *P* = 0.412), which was not preserved when cells were cultured in the absence of rapamycin; *P* < 0.0001 (Figure 3H).

Furthermore, the expression of CXCR3, the chemokine receptor important for the migration and recruitment of Tregs to the liver was measured [27] at baseline and after the 36 days expansion. In the absence of rapamycin a decrease in the Tregs expressing CXCR3 *P* = 0.015 was observed. In contrast, the addition of rapamycin maintained the expression of this marker on the Tregs (Figure 3H). Further analysis detailing the chemokine receptor expression of the final product and the cytokine production of the Tregs is presented in Supplementary Figures 2 and 3.

### *Ex vivo* expanded Tregs from ARC patients have increased suppressive ability

**Figure 4:**
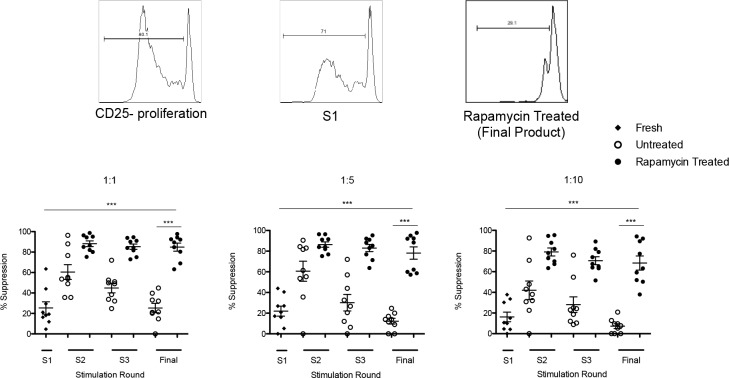
Assessement of Treg suppressive function Representative histogram and graph from 9 ARC patients upon assessment of Treg suppressor function. The suppressive function of Tregs cultured ± rapamycin was assesed by CFSE dilution assay at day 0 and throughout the 36 day culture period. n.s.- not significant. *P < 0.05, ***P < 0.001. Data are represented as mean +/− SEM.

To assess the functional properties of Tregs CFSE dilution assays were performed to evaluate the ability of freshly isolated and *ex vivo* expanded Tregs from patients to suppress the proliferation of T effectors (Figure 4).

The data clearly demonstrated that at the end of expansion, the suppressive function elicited by ARC Tregs in the presence of rapamycin was significantly higher as compared to freshly isolated Tregs at different Treg:Teffector ratios (1:1 ratio - final harvest: 84.8% ± 3.96 *vs*. S1: 25.4% ± 6.03; *P* = *0* .0001, 1:10 ratio -final harvest: 68.3% ± 6.85 *vs*. S1: 16.2% ± 4.68; *P* = 0.0001). In the untreated cultures, despite an increase in Treg suppressive function at day 12 (S2), this increase did not reach similar levels to those obtained from Tregs expanded in the presence of rapamycin (1:1 ratio 60.5% ± 7.26 *vs*. 88.2% ± 2.76, respectively; *P* = 0.0026; and 1:10 ratio 42.0% ± 8.99 *vs*. 79.1% ± 3.90, respectively; *P* = 0.0016). Moreover, the increase in Treg suppressive function was not preserved by final harvest in the untreated cultures as compared to the rapamycin treated cultures (1:1 ratio 25.2% ± 4.58 *vs* 84.8% ± 3.96, respectively; *P* = 0.0001; 1:10 ratio 7.32% ± 2.32 *vs* 68.3 ± 6.85, respectively; *P* = 0.0001) (Figure 4). These results further support the use of the rapamycin based GMP protocol in the expansion of autologous Tregs from ARC patients in view of the increased Treg suppressor function.

### Rapamycin stabilizes the Treg population by preventing IL-17 production

**Figure 5:**
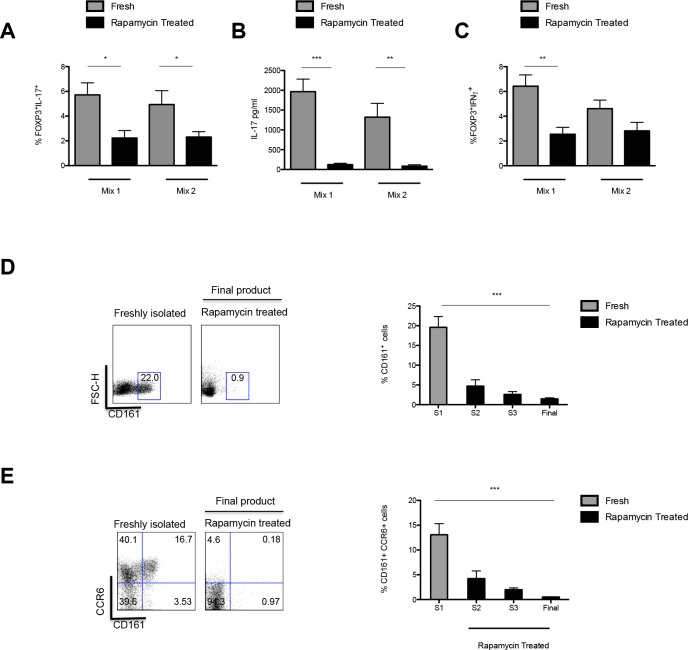
Intracellular expression of IL-17 and IFN-γ and production of IL-17 by *ex vivo* expanded Tregs **A.** Frequency of IL-17^+^ in CD4^+^CD25^+^FOXP3^+^ Tregs upon isolation and post culture in the presence of rapamycin (day 36) when exposed to two separate mixes of pro-inflammatory cytokines (Mix 1: IL-2, IL1β, IL-6 and TGF-β and Mix 2: IL-2, IL-21, IL-23 and TGF-). **B.** IL-17 (pg/ml) concentration in a 5-day culture supernatant of the rapamycin expanded Tregs in the presence of Mix1 and Mix2. **C.** Frequency of IFN-γ^+^ in CD4^+^CD25^+^FOXP3^+^ Tregs upon isolation and post *ex vivo* expansion in the presence of rapamycin (day 36) when exposed to Mix 1 and Mix 2. **D.** Gating strategy and Frequency of CD161^+^ Tregs throughout culture. Dot plot depicts expression of CD161 on CD4^+^CD25^+^ Tregs from a representative sample of 9 ARC patients. Graph shows the dynamics of CD161 expressing Tregs throughout culture. **E.** Gating strategy and Frequency of CCR6^+^CD161^+^ Tregs throughout culture. Dot plot of CD161^+^CCR6^+^ co-expression on CD4^+^CD25^+^ Tregs from a representative sample of 9 ARC patients and graph of percentage CD161^+^CCR6^+^ co-expression throughout culture. n.s.-not significant. *P < 0.05,**P < 0.01. ***P < 0.001. *n* = 9 ARC patients. Data are represented as mean +/− SEM.

One of the major concerns in Treg immunotherapy is the plasticity of Tregs and their conversion to cells producing inflammatory cytokines, when exposed to a pro-inflammatory environment [28, 29]. As such, freshly isolated and expanded Tregs were cultured for 5 days in the presence of Th17 skewing conditions and the percentage of IL-17^+^ cells (Figure 5A) and the production of IL-17 (Figure 5B) assessed by FACS and ELISA, respectively, in order to ascertain their stability. Our data clearly confirmed that over the 36-days culture period the presence of rapamycin resulted in the production of a stable population of Tregs with a reduced percentage of IL-17^+^ cells as compared to baseline, when exposed to a proinflammatory milieu (Mix 1: *P* = 0.0384; Mix 2: *P* = 0.0446). Additionally, diminished IL-17 production was detected on analysis of culture supernatants, confirming the data obtained by intracellular staining (Mix 1: S1 1965pg/ml ± 318 *vs*. final harvest 124pg/ml ± 34.5; *P* < 0.0001; Mix 2: S1 1322pg/ml ± 347 *vs*. rapamycin at final harvest: 84.3pg/ml ± 35.3; *P* = 0.0027) (Figure 5A, 5B). Moreover, assessment of the percentage of IFNγ^+^ cells also clearly demonstrated that rapamycin resulted in a non-inflammatory Treg population with a reduction in the frequency of FOXP3^+^IFNγ^+^ cells by final harvest (Mix 1 S1; 6.42% ± 0.915 *vs*. final harvest 2.54% ± 0.560; *P* = 0.0023; Mix 2; S1 4.61% ± 0.698 *vs*. final harvest 2.82% ± 0.688; *P* = 0.084) (Figure 5C).

To further assess the susceptibility of Tregs to differentiate into Th17 cells the expression of CD161 on the Tregs was investigated. Expression of CD161 by T cells has been reported as a marker for precursors of IL-17 producing T cells and in addition, characterises a population of Tregs with the propensity to produce IL-17, as shown by our group and others [30-33].

Our data showed that during the 36-day expansion period there was a decrease in the percentage of CD161^+^ Tregs (from 19.6% ± 2.42 to 1.48% ± 0.26; *P* < 0.0001) (Figure 5D). In parallel a decrease in the percentage of Tregs co-expressing CD161 and CCR6 in rapamycin treated cultures as compared to baseline was also demonstrated (0.500 ± 0.107 *vs* 12.5% ± 1.75, respectively, *P* < 0.0001) (Figure 5E).

### Tregs from ARC patients were enriched, expanded and cryopreserved in the Clinical Research Facility

To extend the Treg protocol from the laboratory to the GMP facility and validate the process for clinical use in ThRIL, 150ml of peripheral blood was obtained from three patients with ARC and Tregs isolated, using the CliniMACS Plus system and expanded in the BRC CRF, Guy's Hospital. Comparison of the recovery of the isolated cells from ARC patients in the BRC CRF and the research laboratory revealed an initial lower recovery of cells, 3.68 ± 1.18 × 10^6^ *vs*. 7.14×10^6^ ± 1.27, respectively (Figure 1A, Table 1A). Despite this, Tregs from patients were successfully expanded, reaching numbers well over the requirements of the ThRIL trial (Table 1A).

**Table 1A:** Clinical-grade Tregs expanded in the Clinical Research Facility

	Total cells x10^6^			
	S1	S2	S3	Final harvest
ARC 1	1.47	28	172	680
ARC 2	4	17.7	159	1250
ARC 3	5.5	41.3	397.6	2680

All final products reached above the numbers needed for the maximum dose of Treg injection planned (4.5×10^6^) for the ThRIL trial; *n* = 3. Abbreviation: S, stimulation; S1, day 0; S2, day 12; S3, day 24; final, day 36

After *ex vivo* expansion, a comprehensive analysis of the Tregs was performed to ensure the final product satisfied the specified release criteria, essential for their clinical application (Supplementary Table 4). At final harvest the mean percentage of cells with a CD4^+^CD25^+^FOXP3^+^ phenotype was 89.2% ± 2.61 with 0.913% ± 0.591 CD8^+^ cells, with a mean viability of 94.9% ± 1.98. Evaluation of the potency of the final product showed the expansion of Tregs with suppressive ability >65% (Table 1B).

Of note, however, ThRIL requires the final product to be injected 3 months post transplantation and therefore necessitates cryopreservation of these cells. Following cryopreservation >80% live cells were recovered and the results demonstrate that Tregs maintained their phenotype and function (Table 1B).

**Table 1B:** Treg manufacture in the Clinical Research Facility

Test	Specification	ARC 1		ARC 2		ARC 3	
		Final harvest	Defrosting	Final harvest	Defrosting	Final harvest	Defrosting
**Months post cryopreservation**			3		3		3
**Identity:**	**Positive for CD4 CD25 FOXP3**	YES	YES	YES	YES	YES	YES
**Purity:**	**≥ 60% of entire cell population CD4**^+^**CD25**^+^ **FOXP3**	93.9	89.1	88.8	92.6	84.9	97.0
**Impurity:**	**≤ 10% CD8**	0.36	1.14	0.43	0.5	1.95	0.6
	**≤100 beads per 3×10**^6^**cells**	75	-	23	-	45	-
**Viability:**	**≥ 70% viability**	91.0	93	97.3	85.0	96.5	84.0
**Recovery (live):**	**≥ 70%**	-	95	-	80.0	-	114
**Potency:**	**≥ 60% suppression**	93.0	99	65.0	97.0	-	95.0
**Safety tests:**	**Sterility-no growth**	No growth	-	No growth	-	No growth	-
	**Endotoxin ≤175IU/mL**	<5 IU/ml	-	<5 IU/ml	-		-
	**Mycoplasma-not detected**	not detected	-	not detected	-		-

All final products met the necessary release criteria on day 36 for their clinical application. After 3 months post cryopreservation cells maintained their phenotype and suppressive function.

Abbreviation: ARC, alcohol related cirrhosis

## DISCUSSION

One of the major drawbacks following liver transplantation is the requirement for lifelong treatment with immunosuppressants with their accompanying side effects and complications. This contributes to a disappointing 60% patient survival rate at 10 years after transplantation. In this regard, the use of cell-based therapies, harnessing the natural immunoregulatory properties of the immune system, is an emerging therapeutic option and Tregs have been recognized as ideal candidates in this endeavor.

ThRIL is a pioneering two stage clinical trial investigating the use of autologous Treg immunotherapy as an individualised medicine to promote transplantation tolerance in liver transplantation. Stage I, prioritises the safety and tolerability of Treg immunotherapy with Treg efficacy assessed in Stage II whereby immunosuppression withdrawal will be attempted in transplant recipients who have received the highest tolerated dose of Tregs.

To date studies aimed at withdrawing immunosuppression in liver transplant recipients early post transplantation have been largely unsuccessful [34] highlighting the need for an intervention, such as that proposed in ThRIL, to promote ‘tolerance’.

Here, we outline the first account of the *ex vivo* expansion of Tregs from prospective liver transplant recipients at GMP standards. The data presented describes the clinical grade isolation strategy and demonstrates the feasibility of autologous Treg cell-based therapy in liver transplantation.

We present data on the selection of a pure population of Tregs (CD4^+^CD25^+^ at S1: 77.7% ± 10.3), using a two-step magnetic activated cell sorting (MACS) protocol. In two of the reported clinical trials [13, 16] a higher degree of Treg purity was attained using Treg isolation based on the combined expression of CD4^+^, CD25^+^, and low expression of CD127 molecules [22]. Whilst cell isolation based on a combination of these markers is highly effective, the lack of GMP cell sorter facilities in the UK makes this translationally unfeasible. In addition, Marek et al. showed that during the expansion process Tregs were “transforming” into effector/memory like cells and proposed that regardless of the phenotypic markers used for Treg isolation, the only variable to maintain Treg phenotype and function is to limit the duration of expansion to 2 weeks [35].

In support of this study, others have also shown that the large-scale manufacture of Tregs remains challenging, reporting that even when starting with a highly pure population of Tregs, repeated stimulation results in the loss of FOXP3 expression [21]. In the clinical trial conducted by Trzonkowski et al. a decrease in the percentage FOXP3^+^ cells after successive weekly stimulation was reported [13]. However, the disadvantage of limiting cultures to two rounds of stimulation became evident in the trial of Treg immunotherapy in Type 1 diabetes where the authors noted an insufficient Treg yield in four out of the ten patients [16].

We generated Tregs in concordance with the proposed GMP protocol, expanding these cells *in vitro* for 36 days with three rounds of stimulation, in the presence of rapamycin. This resulted in the expansion of Tregs to numbers sufficient for administration of the maximum dose for the ThRIL trial. Despite the three rounds of stimulation, and contrary to the studies outlined above, FOXP3 expression was maintained in culture and the data clearly demonstrated that in the presence of rapamycin there was an increase in the percentage of FOXP3^Hi^ Tregs and in the level of expression of FOXP3 at final harvest (Figure 3B, 3C).

One of the concerns with the clinical translation of bead-separated Treg preparations is the presence of ‘contaminating’ effector T cells. However, as has been reported previously [18], expansion of Tregs in the presence of rapamycin led to the enrichment of a Treg population with increased CD127lo and CTLA-4 expression (Figure 3D, 3F), correlating with the pronounced increase in suppressive function (Figure 4). CTLA-4 has been recognized as an important regulatory marker expressed by Tregs and has been reported to play an important role in their suppressive function, through the internalization of co-stimulatory molecules on antigen presenting cells (APC) [23]. This has significant implications, concerning the clinical application of autologous patient derived Tregs in the setting of liver transplantation, whereby our protocol ensures the high expression of this key regulatory molecule.

Another major potential barrier to Treg therapy is the possibility that these cells may assume a pro-inflammatory phenotype. In this study, we provide further evidence to our published data where we report that conditioning of Tregs with rapamycin leads to the production of a stable, non-inflammatory Treg population due to the inhibition of IL-17 and IFN-γ production by Tregs *in vitro* in the presence of intense inflammatory conditions (Figure 5A, 5B) [18, 36].

In line with this, a decrease in the percentage of cells co-expressing CD161 and CCR6 was demonstrated, confirming previous work [37]. Moreover, in view of the plasticity of Tregs, Kopft et al. studied the reciprocal differentiation of Tregs and Th17 cells and reported that rapamycin suppresses the differentiation of pathogenic Th17 cells [38]. As such, patients in the ThRIL study will be taking rapamycin by the time of Treg infusion. This adjunctive immunosuppression has the dual advantages of promoting Treg survival and stability by reducing the risk of the injected cells acquiring pro-inflammatory features.

Furthermore there have been reports of the importance of epigenetic control of Foxp3 expression through the methylation of the Treg specific demethylated region (TSDR). Future research will be focused at understanding Treg commitment and epigenetic regulation of FOXP3 expression so that the mechanisms can be harnessed to stabilise the Tregs, of importance for their clinical application.

In addition to stability, therapeutic strategies using Tregs have to also take into account the need for appropriate tissue trafficking to enable contact with their target cells. The expression of the chemokine receptor, CXCR3, has been reported to mark subsets of T cells associated their migration to sites of inflammation [39]. Furthermore, in a murine model of T cell mediated liver injury, Lapierre et al. demonstrated the ability of CXCR3^+^ Tregs to migrate to the liver, in turn potentiating the effectiveness of Treg adoptive transfer [40]. The data presented here demonstrates the preservation of cells expressing CD62L and CXCR3, important for their passage to lymph nodes and the liver respectively (Figure 3H).

Finally, we show that despite a lower recovery of isolated cells (Tables 1A & 1B), partly explained by the closed system of isolation procedure which involves a longer, more vigorous cell isolation procedure, adequate numbers of Tregs, that meet the trial release criteria, can be expanded in the BRC CRF at Guy's Hospital and remain functional after freeze-thawing, a prerequisite precluding their clinical translation.

Additionally, it is pertinent to note that initial confidence in adoptive Treg cell therapy as a self-sufficient entity, experimental data has shown that the efficacy of Treg therapy requires the setting of a favorable *in vivo* environment, supporting both the cell engraftment and the chance of inducing tolerance, such as transient host T cell depletion by immunosuppressive treatments [41, 42]. This highlights the importance of strategies to tailor immunosuppressive therapy to ensure the *in vivo* survival of the injected Tregs or enhance their longevity *in vivo*. In this regard the clinical protocol for ThRIL is based on a Treg supportive immunosuppressive regimen including the use anti-thymocyte globulin (ATG), to induce lymphopenia with a preferential preservation of Tregs [43]. Additionally to limit memory T cell expansion post ATG induction, patients are started on tacrolimus and prednisolone and a month prior Treg infusion maintained on low dose tacrolimus with the addition of rapamycin, to promote selective Treg expansion *in vivo* [44]. The intention behind this protocol: to create a tolerogenic milieu thus maximizing the potential efficacy of the exogenously administered Tregs through prolongation of their *in vivo* survival. It is also reassuring that these cells will be injected in a ‘Treg nurturing’ environment, centered on the inclusion of rapamycin, as compared to other immunosuppressants whose indiscriminant mechanism of action poses a threat to Treg survival.

Thus, tailoring the immunosuppressive regimen along with the administration of *ex vivo* expanded Tregs may potentially maintain post liver transplant tolerance, accomplishing the ultimate aim of Treg immunotherapy trials in this setting.

Presently, the first two patient recruited into the ThRIL trial have received the first Treg injection, with no reported toxicity to date. The near future will now see the reporting of this clinical trial along with the much anticipated immunomontoring data, informing the *in vivo* dynamics of the cells expanded. There is no question that the information gleaned from the ThRIL trial will serve as to guide the transition of Tregs as a viable therapeutic option in liver transplant recipients.

Additionally, with the advent of GMP-compliant FACS sorting there will be developments in the current GMP protocol for the manufacture of the final cell product with a focus on the optimal Treg subset with potent suppressive function, specificity, and those that are epigenetically stable.

## MATERIAL AND METHODS

### Participant selection criteria

150ml of peripheral blood was obtained from 12 patients with alcohol related cirrhosis (ARC), awaiting transplantation. Patient selection was based on the inclusion criteria in accordance with the ThRIL clinical protocol (NCT02166177) (Supplementary Table 1 and Table 2). As outlined by the UK transplant registry http://www.odt.nhs.uk/uk-transplant-registry/ cirrhosis secondary to alcohol is one of the leading indications for liver transplantation in the UK and as such this cohort of patients form the majority of patients recruited into the ThRIL trial.

Peripheral blood was obtained from 9 age and sex matched healthy controls (HCs). All blood samples were handled and disposed of in concordance with Human Tissue Act 2008. Human studies were conducted in accordance with the Declaration of Helsinki and approved by the Institutional Review Board (09/H0707/86).

### Treg isolation

In the laboratory, peripheral blood mononuclear cells (PBMC) were isolated from patients and HCs by lymphocyte (PAA, Pasching, Austria) density gradient centrifugation and depleted for CD8^+^ cells followed by enrichment of CD25^+^ T cells using magnetic beads (Miltenyi Biotec, Woking, UK) and as previously reported [17]. All reagents and consumables used were of clinical GMP grade.

In the BRC CRF, blood volume was reduced, using the Sepax^®^ 2 device (Biosafe) prior to Treg isolation. Tregs were purified using a combination of CD8^+^ reduction (CliniMACS CD8 reagent, Miltenyi Biotec, Woking, UK) and enrichment steps for CD25^+^ cells (CliniMACS CD25 reagent, Miltenyi Biotec), using the automated CliniMACS^®^ Plus System (Miltenyi Biotec) in the BRC CRF at Guy's Hospital. All processing steps were performed in closed systems, using single use tubing sets.

### Expansion of Treg lines

Isolated cells were plated at 1×10^6^/ml cell and activated with anti-CD3/CD28 coated beads (Invitrogen, Paisley, UK; Miltenyi Biotech) at a 2:1 bead:cell ratio. Cells were expanded in culture media X-vivo 15 (Lonza, Basel, Switzerland) 5% human AB serum (HS) (Biosera, Ringer, UK; Lonza) containing rapamycin (100 nM) (Rapamune^®^, Wyeth, USA) for 36 days. IL-2 (500 IU/ml, Proleukin^®^, Novartis, UK) was added at day 4 post-activation and replenished every 2 days. Cells were restimulated every 10-12 days. Beads were magnetically removed and the cells washed in PBS. This process was repeated to ensure bead removal following which, fresh beads, rapamycin and IL-2 were added. Expanded cells were used for further analysis at each time of restimulation up until day 36 of expansion.

In the BRC CRF, under GMP conditions, enriched cells were seeded in MACS^®^ GMP Cell Expansion Bags at 0.5×10^6^ cells/mL in TexMACS™ GMP Medium (Miltenyi Biotec) supplemented with 5% human AB serum (Seralab, UK), containing 100 nM rapamycin (Rapamune^®^, Pfizer) and activated with anti-CD3/CD28-coated beads (4:1 bead:cell ratio, MACS GMP ExpAct Treg Kit, Miltenyi Biotec). Human recombinant IL-2 (500 IU/mL; Proleukin^®^, Novartis) was added at day 4-6 and replenished every 2-3 days. The cells were rested 4 days before restimulation. Stimulation occurred on days 12 and 24, during which time cells were pooled, fresh beads (1:1), rapamycin, and IL-2 added. At final harvest, the pooled cells were processed on the CliniMACS Plus Instrument using a pre-set depletion program to remove the ExpAct Treg expansion beads. A sample of the cells was assessed for safety and functional analysis.

### Flow cytometry

Flow cytometric analysis was performed on freshly isolated and expanded Tregs using the BD FACSCanto™ cell analyzer (BD Bioscience, Oxford, UK) and analysed using FlowJo software *(TreeStarInc, OR, USA).* In short, cells were washed and stained with the listed mAbs in the Supplementary Table 3 for 30 min at 4°C. Appropriate isotype control antibodies were used for each sample. Following staining, cells were examined by flow cytometry.

Intracellular staining for FOXP3 was performed in accordance with the manufacturer's protocol (eBioscience). Expression levels of and IL-17 was assessed after activation of cells with phorbol myristate acetate (PMA, 5ng/ml, Sigma Aldrich, St Luis, MO, USA), Ionomycin (1μg/ml, Sigma Aldrich, St Luis, MO, USA) and Monensin (2μM, eBioscience, San Diego, CA, USA) for 4 hours. Subsequently, the intracellular staining for IL-17 was performed according to the manufacturer's protocol.

In the BRC CRF, flow cytometry, using CD4-PerCP/Cy™5.5, CD-25PE, CD8-APC was carried out on the BD FACSCanto™ cell analyzer (BD Bioscience). Intracellular staining for FOXP3-FITC was performed, as above, in accordance with manufacturer's protocol (eBioscience). Appropriate isotype controls and Fluorescence minus one controls were used to assign gates and analysis carried out, using the FlowJo software.

### Suppression assay

Responder CD4^+^CD25^−^ T cells were obtained from PBMCs by negative selection using unconjugated anti-CD8 (6μL/10^8^cells), anti-CD33 (3μL/10^8^cells) (both Caltag, California, USA), anti-CD14 (7μL/10^8^cells), anti-CD16 (7μL/10^8^cells), anti-CD19 (6μL/10^8^cells), anti-CD56 (3μL/10^8^cells), anti-γδ TCR (7μL/10^8^cells) and glycophorin CD235a (all Diaclone, Gen-probe, San Diego, USA) antibodies with pan-IgG microbeads and anti-CD25 microbeads (both Invitrogen, Paisley, UK)

Aliquots of the CD4^+^CD25^−^ cells were cryopreserved and used as allogeneic responder cells in suppression assays.

Cryopreserved responder CD4^+^CD25^−^ T cells (Teff) were thawed and labelled with 2.5 nM the carboxyfluorescein succinimidyl ester (CFSE) (Molecular Probes, Carlsbad, CA, USA). Responder purity was >95% (Supplementary Figure 4). 1×10^5^/well of responder T cells were co-cultured at different ratios (Treg: Teff = 1:1, 1:5 and 1:10) with Tregs in X-Vivo 15 medium supplemented with 5% HS and activated by anti-CD3/CD28-coated beads (Invitrogen, Paisley, UK) in U-bottom 96-well plates. Cells were incubated at 37°C, 5% CO_2_ for 5 days. After harvest, proliferation of CFSE-labelled responder cells was determined by flow cytometry (FACSCalibur or on *LSRFortessa™ cell analyzer (BD Bioscience)* and analyzed with FlowJo software (Tree Star Inc, OR, USA). The suppressive ability of Treg lines was assessed as the percentage decrease of Teff proliferation in the presence of Tregs. The calculation was based on the proliferation of responder T cells alone compared with the proliferation of cultures also containing Treg cells.

### Treg culture in the presence of pro-inflammatory cytokines

Freshly isolated, untreated and rapamycin treated CD4^+^CD25^+^ T cells (5×10^5^) were activated with anti-CD3/CD28 coated beads at 1:1 bead:cell ratio and cultured for 5 days in the presence of pro-inflammatory cytokine cocktails: Mix 1: IL-2 (10 IU/ml), IL1β (10ng/ml, R&D Systems, Minneapolis, MN, USA), IL-6 (4ng/ml, R&D Systems, Minneapolis, MN, USA) and TGF-β (5ng/ml, R&D Systems, Minneapolis, MN, USA). Mix 2: IL-2 (10 IU/ml), IL-21 (25ng/ml, Cell Sciences, Canton, MA, USA), IL-23 (25ng/ml, R&D Systems, Minneapolis, MN, USA) and TGF-β (5ng/ml, R&D Systems, Minneapolis, MN, USA). Cells cultured in complete medium supplemented with IL-2 (10 IU/ml) were used as controls to ensure their survival throughout the 5-day stability assay.

At the end of the culture cells were harvested, activation beads removed by magnetic adherence and analysed for IL-17 and IFN-γ expression by intracellular staining. IL-17 in supernatants was analysed by an indirect sandwich ELISA.

### ELISA

ELISA for human IL-17 was carried out using the Duo-Set ELISA kit from R&D (Abingdon, UK) according to manufacturer's instructions

### Cryopreservation of the expanded Tregs in the CRF

After final harvest, all batches were assessed against the set release criteria for the ThRIL trial (Supplementary Table 4) and subsequently cryopreserved. In brief, Treg numbers equivalent to doses for the ThRIL trial were resuspended in 2.1ml of CryoStor^®^ CS10 freezing media, transferred to a CellSeal^®^ Cryovial and placed in a controlled rate freezer before transfer to liquid nitrogen (vapor phase) for long-term storage.

In order to assess the recovery of the cryopreserved product and the effect of cryopreservation on the biology and function of the final product, cells were thawed, diluted in 5% human serum albumin, and the viability and suppressive function of the cryopreserved product was assessed.

### Statistical analysis

Statistical analysis was carried out on GraphPad Prism 5.0c (GraphPad software Inc. CA, USA). Parametric and nonparametric data were expressed as mean ± standard error and median where appropriate. For comparison of parametric data, paired and unpaired students t-tests were used. Statistical significance was set at *P* < 0.05.

*P< 0.05, ** *P*< 0.01 and *** *P*< 0.001

## SUPPLEMENTARY MATERIAL FIGURES AND TABLES


