# Supplementary material for: Successful expansion of functional and stable regulatory T cells for immunotherapy in liver transplantation
Source: Oncotarget. 2016 Jan 17;7(7):7563–77. doi: 10.18632/oncotarget.6927 (PMC4884938; doi:10.18632/oncotarget.6927)
Supplement: Supplementary file 1 [file oncotarget-07-7563-s001.pdf]

Successful expansion of functional and stable regulatory T cells for immunotherapy in liver transplantation

Supplementary Material

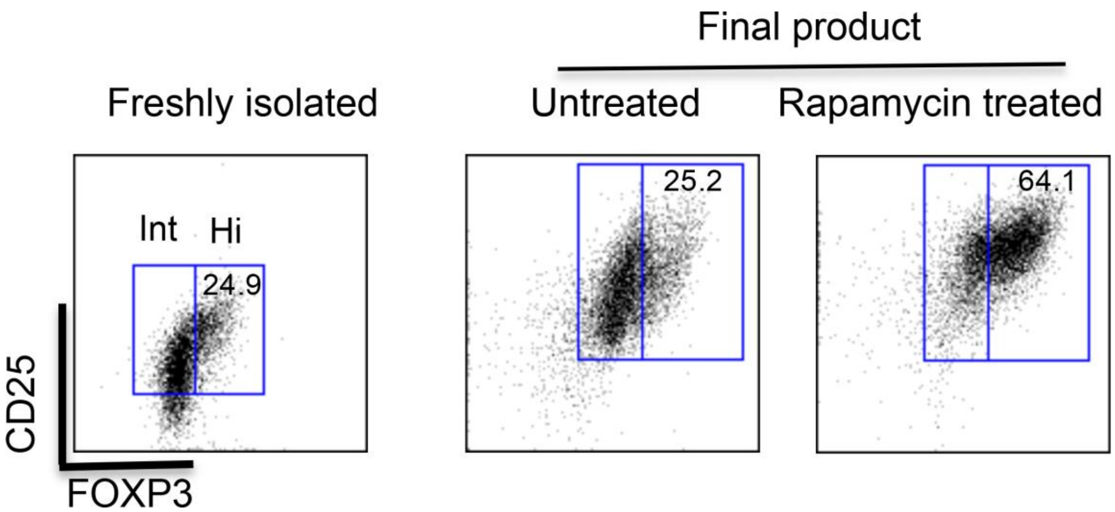

Supplementary Figure 1: FOXP3 differential gating strategy

A representative flow cytometric plot depicting the gating strategy applied to delineate a population with a high expression of FOXP3 (Hi) and one with intermediate expression of FOXP3 (Int).

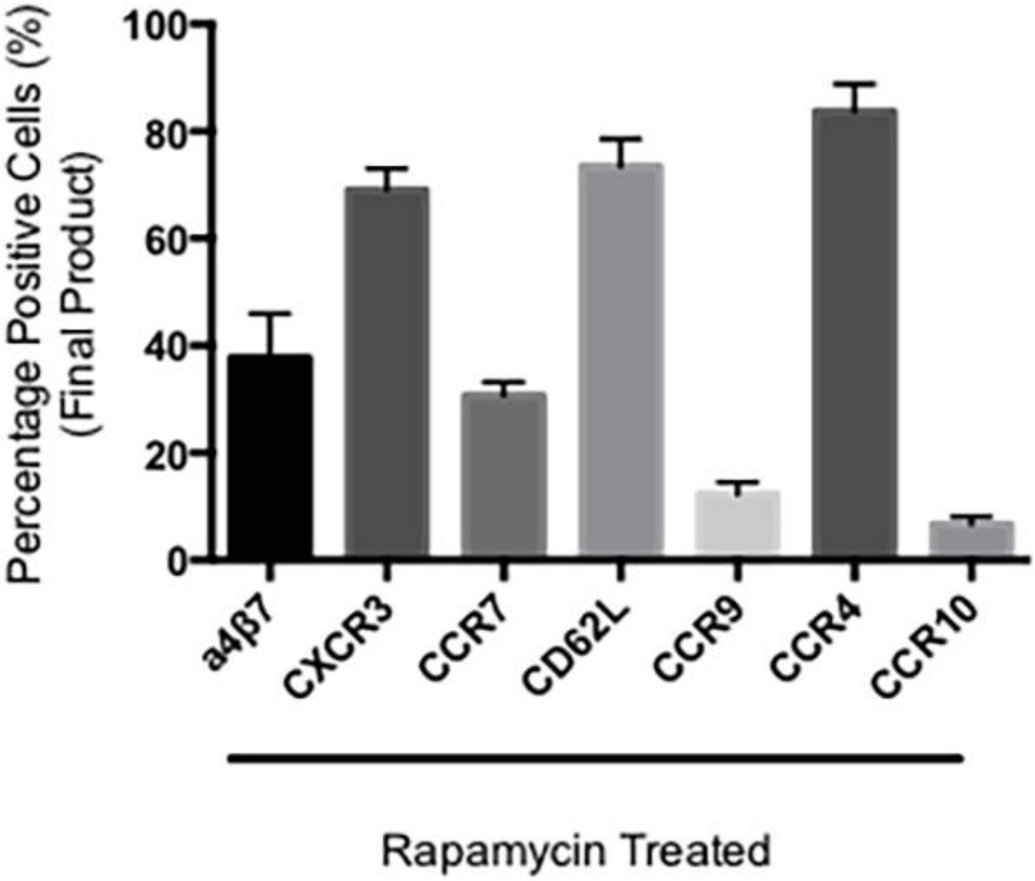

Supplementary Figure 2: Chemokine receptor expression

A graph showing various CD4+CD25+ Treg chemokine receptors expression at final harvest (day 36).

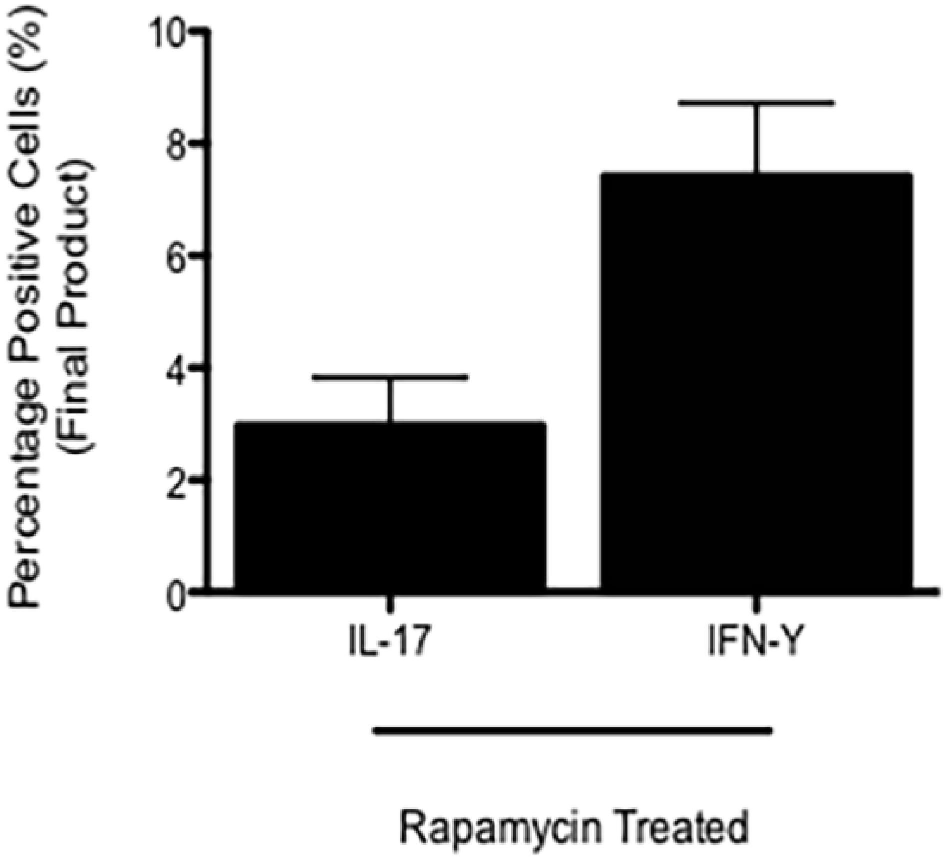

Supplementary Figure 3: Cytokine expression of final product at baseline

A graph showing the relative expression of IL-17 and IFN-y on CD4+CD25+ Tregs at final harvest after 36 days of treatment with rapamycin, following 5hour exposure to PMA, ionomycin and monensin.

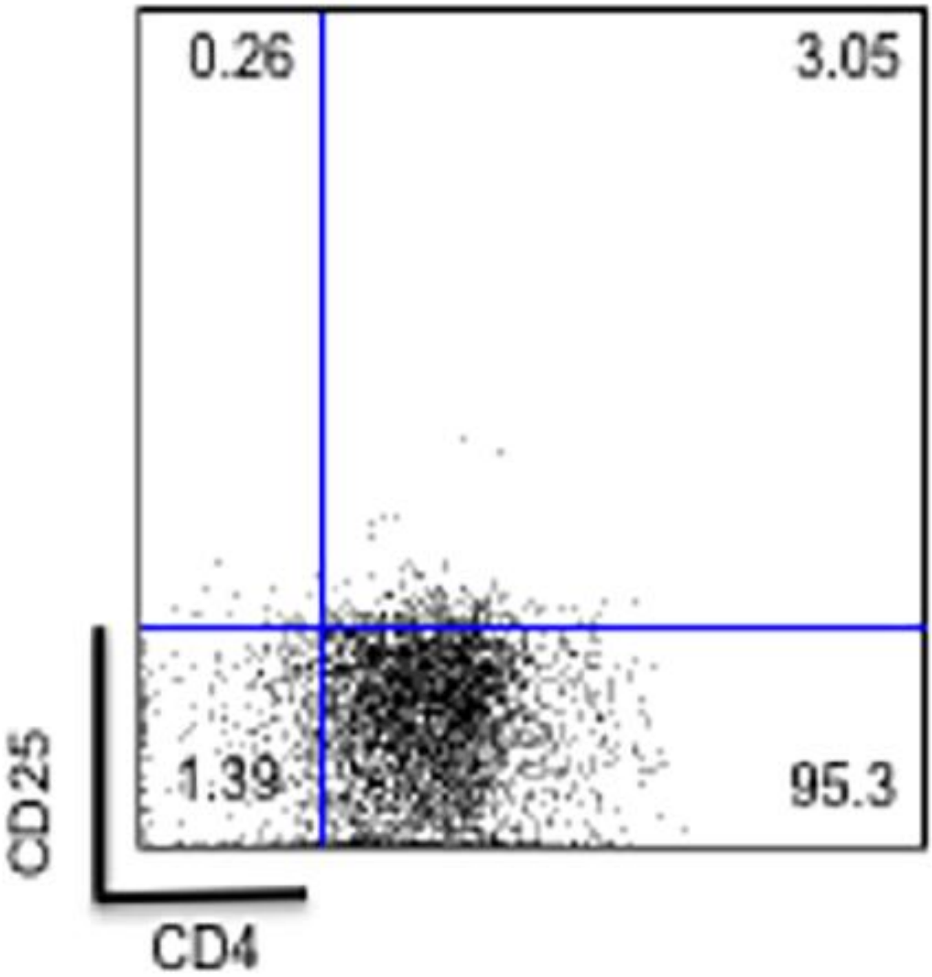

Supplementary Figure 4.

A representative flow cytometric plot depicting purity of Effector CD4+CD25- Cells.

.

Supplementary Table 1: Subject Demographics

	**ARC**	**HC**
**Total Number**	12	9
**Sex (Male:Female)**	10:2	7:2
**Age (mean± SD); years**	54.1 ± 10.9	50.7 ± 10.1
**Average MELD score**	13.3 ± 3.56	N/A

Tregs from 12 Patients with ARC and on the liver transplant waiting list were recruited in to the study. Patient Inclusion/exclusion criteria as per ThRIL (NCT02166177). Inclusion Criteria: able to give informed consent, adult patients with end-stage liver disease listed for primary liver transplant, calculated MELD score ≤ 25 at time of transplantation; Exclusion Criteria: HIV or RNA-positive Hepatitis C Virus infection; autoimmune liver disease, previous organ transplant, Ebstein Virus and/or Cytomegalovirus sero-negativity, chronic use of systemic immunosuppressants, hepatocellular carcinoma outside Milano criteria, leukocytes <1.5x10^9/L and/or platelets <50x10^9/L.

9/12 patient samples were isolated and expanded in the research laboratory and data compared to age and sex matched healthy control (HCs). Tregs from 3/12 ARC patients were manufactured in the GMP Facility. ARC = alcohol related liver disease; HC= healthy control; N/A= not applicable; MELD: Model for End-Stage Liver Disease.

Supplementary Table 2: Patient Laboratory data

	Alcohol-Related Cirrhosis Patients
**Number of Patients**	9
**Hb (nv: 115-180g/l)**	120 (83.0-144)
**Platelets (nv: 150-400x10^{9}/l)**	93.5 (39.0-415)
**INR (nv: 0.9-1.1)**	1.53 (1.04-3.31)
**Bilirubin (nv: <20μmol/l)**	40.5 (7.00-137)
**ALP (nv: 40-165U/l)**	120 (46.0-242)
**AST (nv: <50U/l)**	42.0 (19.0-94.0)
**γGT (nv: <60U/l)**	51.5 (17.0-365)
**Albumin (nv: 35-50g/l)**	33.5 (23.0-42.0)
**Creatinine (nv: 50-120μmol/l)**	76.0 (33.0-135)

Data are presented as the median (range). Abbreviations: ALP- Alkaline phosphatase; AST- Aspartate transaminase; γGT- gamma glutamyl transferase; INR- International Normalised Ratio; nv- normal values.

Supplementary Table 3: Antibodies used for flow cytometry

Specificity	Conjugate	Clone	µL/10^6cells	Supplier	
**CCR6**	PeCy7	R6H1	3	eBioscience	San Diego, USA
**CD4**	PercP	SK3	5	BD Bioscience	Oxford, UK
**CD8**	PeCy7	SK1	5	eBioscience	San Diego, USA
**CD25**	APC	2A3	5	BD Bioscience	Oxford, UK
**CD25**	PE	CD25-4E3	3	eBioscience	San Diego, USA
**CD27**	eFluor450®	O323	2	eBioscience	San Diego, USA
**CD39**	PeCy7	eBioA1	2	eBioscience	San Diego, USA
**CD62L**	PeCy7	DREG-56	2	eBioscience	San Diego, USA
**CD127**	eFluor450®	eBioRDR5	2	eBioscience	San Diego, USA
**CD161**	eFluor450®	HP-3G10	3	eBioscience	San Diego, USA
**CTLA-4 (CD152)**	PE	14D3	3	eBioscience	San Diego, USA
**CXCR3**	Pacific Blue	G025H7	3	Biolegend	San Diego, USA
**FOXP3**	FITC	236A/E7	5	eBioscience	San Diego, USA
**FOXP3**	PE	236A/E7	5	eBioscience	San Diego, USA
**HLA-DR**	PeCy7	LN3	2	eBioscience	San Diego, USA
**IFN-γ**	PeCy7	4S.B3	1.5	eBioscience	San Diego, USA
**IL17**	PE	eBio64DEC 17	2	eBioscience	San Diego, USA

APC, allophycocyanin; FITC, fluorescein isothiocyanate; PE, Phycoerythrin; PerCP, Peridinin-Chlorophyll-Protein Complex;

Supplementary Table 4: Release criteria for the clinical application of the final product in the ThRIL trial.

Test	Specification
**Purity**	≥ 60% of entire cell population $CD4^+$ $CD25^+$ FoxP3
**Impurities**	≤10 % CD8<br>≤100 beads per $3x10^6$ cells<br>Viability ≥70%
**Contaminants**	Sterility- no growth after 5 days<br>Endotoxin- ≤175 IU/ml<br>Mycoplasma - not detected
**Potency**	≥60% suppression
